# Evaluating Factors that Increase Research Study Matriculation

**DOI:** 10.1002/alz70858_101108

**Published:** 2025-12-25

**Authors:** Reginald Wilburn, Le'Elle Davis, Theresa F. Gierzynski, Jennifer Dinh, Kate Hanson, Holly Bunker, Bernardo Flores, Joshua T Fox‐Fuller, Renee Gadwa, Arijit K Bhaumik, J. Scott Roberts, Henry L Paulson, Annalise Rahman‐Filipiak

**Affiliations:** ^1^ Michigan Alzheimer's Disease Research Center ‐ University of Michigan, Ann Arbor, MI, USA; ^2^ Michigan Alzheimer's Disease Research Center, Ann Arbor, MI, USA

## Abstract

**Background:**

In the Alzheimer's Disease Research Center (ADRC) network and beyond, registries are commonly established to support representative recruitment into longitudinal cohorts and affiliated studies. While significant attention has focused on how to recruit potential participants into registries, less is known about how to promote registry engagement including matriculation into affiliated studies and attendance at research events. This study aims to identify the barriers and facilitators to participating in Alzheimer's disease (AD) research among diverse older adults in a longstanding AD research registry.

**Method:**

An electronic survey was sent to all participants in the Michigan ADRC MiNDSet research registry between November and December 2024. 540 participants (age=71.7±7.7, education = 16.6 ±2.2) responded, 67.2% of whom self‐identified as female, 77 % of whom identified as white, 14% of whom identified as Black, and 1.3% of whom identified as Asian. Survey items explored demographic and social factors, reasons for signing up for the registry, and barriers versus facilitators to engaging in studies to which registry members had been referred.

**Result:**

The most reported deterrents to enrolling in studies were conflicts of schedule (*n* = 54) and location of visit (*n* = 47), while lack of trust (*n* = 2) or personal benefit (*n* = 12) did not deter participants. 63.7 % of respondents endorsed having a positive experience in research, with staff empathy, staff competency and the opportunity for feedback identified as thematic incentives in participant free responses. The potential for feedback was the most identified factor contributing to positive participant experience. 97 % of participants were interested in future studies, including Education and Support studies (60%), Diagnostic and Feedback studies (59%), Genetic Treatments (54%), and Non‐Pharmacologic Trials (54%).

**Conclusion:**

While the sample represents those most engaged in the MADRC registry, preliminary results highlight several tangible targets for studies wishing to recruit diverse older adults into AD research. Logistical flexibility around location and timing of visits may reduce burden and thereby increase willingness to engage in studies. The degree of preparation, organization, communication, and empathy of the study staff can serve as facilitators to engagement. The opportunity to learn individual research results can greatly enhance engagement in research.

